# Homocysteine Lowering by Folate-Rich Diet or Pharmacological Supplementations in Subjects with Moderate Hyperhomocysteinemia

**DOI:** 10.3390/nu5051531

**Published:** 2013-05-08

**Authors:** Bruno Zappacosta, Pierpaolo Mastroiacovo, Silvia Persichilli, George Pounis, Stefania Ruggeri, Angelo Minucci, Emilia Carnovale, Generoso Andria, Roberta Ricci, Iris Scala, Orazio Genovese, Aida Turrini, Lorenza Mistura, Bruno Giardina, Licia Iacoviello

**Affiliations:** 1 Department of Laboratory, Foundation of Research and Care, “John Paul II”, Catholic University of Sacred Heart, Campobasso 86100, Italy; 2 ICBD, Alessandra Lisi International Centre on Birth Defects and Prematurity, Rome 00100, Italy; E-Mail: icbd@icbd.org; 3 Department of Laboratory Medicine, Policlinico A. Gemelli, Catholic University of Sacred Heart, Rome 00168, Italy; E-Mails: spersichilli@rm.unicatt.it (S.P.); aminucci@rm.unicatt.it (A.M.); bgiardina@rm.unicatt.it (B.G.); 4 Research Laboratories, Foundation of Research and Care, “John Paul II”, Catholic University of Sacred Heart, Campobasso 86100, Italy; E-Mails: gdpounis@moli-sani.org (G.P.); licia.iacoviellooli-sani.org (L.I.); 5 National Institute of Research for Foods and Nutrition (Istituto Nazionale di Ricerca per gli Alimenti e la Nutrizione), Rome 00178, Italy; E-Mails: ruggeri@inran.it (S.R.); carnovale@inran.it (E.C.); turrini@inran.it (A.T.); mistura@inran.it (L.M.); 6 Department of Pediatrics, Federico II University, Neaples 81100, Italy; E-Mails: andria@unina.it (G.A.); scala@unina.it (I.S.); 7 Department of Pediatrics, Catholic University of Sacred Heart, Rome 00168, Italy; E-Mails: rricci@rm.unicatt.it (R.R.); ogenovese@rm.unicatt.it (O.G.)

**Keywords:** folic acid, 5-MTHF, homocysteine, folate-rich diet, MTHFR genotype, clinical trial

## Abstract

**Background/Objectives:** To compare the efficacy of a diet rich in natural folate and of two different folic acid supplementation protocols in subjects with “moderate” hyperhomocysteinemia, also taking into account C677T polymorphism of 5,10-methylenetetrahydrofolate reductase (MTHFR) gene. **Subjects/Methods:** We performed a 13 week open, randomized, double blind clinical trial on 149 free living persons with mild hyperhomocyteinemia, with daily 200 μg from a natural folate-rich diet, 200 μg [6*S*]5-methyltetrahydrofolate (5-MTHF), 200 μg folic acid or placebo. Participants were stratified according to their MTHFR genotype. **Results:** Homocysteine (Hcy) levels were reduced after folate enriched diet, 5-MTHF or folic acid supplementation respectively by 20.1% (*p* < 0.002), 19.4% (*p* < 0.001) and 21.9% (*p* < 0.001), as compared to baseline levels and significantly as compared to placebo (*p* < 0.001, *p* < 0.002 and *p* < 0.001, respectively for enriched diet, 5-MTHF and folic acid). After this enriched diet and the folic acid supplementation, Hcy in both genotype groups decreased approximately to the same level, with higher percentage decreases observed for the TT group because of their higher pre-treatment value. Similar results were not seen by genotype for 5-MTHF. A significant increase in RBC folate concentration was observed after folic acid and natural folate-rich food supplementations, as compared to placebo. **Conclusions:** Supplementation with natural folate-rich foods, folic acid and 5-MTHF reached a similar reduction in Hcy concentrations.

## 1. Introduction

Several large-scale epidemiological studies, mainly retrospective ones, have lead, during the last three decades, to the appreciation of the sulfur-containing aminoacid homocysteine (Hcy) as an independent risk factor for cardiovascular disease, mainly coronary heart disease and stroke, although its causal effect has been recently challenged by some B-vitamins Hcy-lowering trials [1], Hcy has been involved in the occurrence of other disorders such as cognitive disease [2,3] cancer [4,5] and, overall, adverse outcome of pregnancy and congenital defects [6,7].

Plasma levels of Hcy are closely dependent on nutritional and genetic factors: the B-vitamins folate, vitamin B12 and vitamin B6 are required for Hcy metabolism; suboptimal folate status, together with the other B-vitamins deficiency, is commonly associated with elevated plasma Hcy, as a consequence of the role that folate plays in the one-carbon metabolism. Among the main determinants of Hcy levels, especially in the presence of low folate concentrations, we have to mention some genetic polymorphisms, mainly the C677T of the enzyme 5,10-methylenetetrahydrofolate reductase (MTHFR): the TT genotype is frequently associated with elevated plasma levels of Hcy, due to a reduced production of 5-methyltetrahydrofolate (5-MTHF).

With regard to the present knowledge, the best strategy to ensure an optimal folate status is still uncertain; this is due to variable dietary habits, variable folate intake with diet, variable prevalence of mutated allele of the MTHFR gene in the different populations studied and the different bioavailability of natural folate and synthetic folic acid [8]. Recently, Dhonukshe-Rutten *et al.* [9] have shown how, in several European countries, both the intake and the B-vitamins status are inadequate and the Hcy concentration higher than ideal.

In Italy, a country in which a mandatory food fortification with folic acid has not been implemented and the use of fortified food is quite limited, the wide availability of folate-rich foods could suggest as the better strategy a folate-rich diet compared to daily intake of synthetic products as capsules. Furthermore, due to the relative high frequency of polymorphisms in MTHFR gene in the Italian population, it could be useful to verify the real efficacy of a greater folate intake in homozygote and heterozygote subjects for the mutated MTHFR gene, in reducing plasma levels of homocysteine.

Aim of this study was to compare the efficacy of a diet particularly rich in natural folate in relation to that of two different protocols of folic acid pharmacological supplementation, in subjects with “moderate” hyperhomocysteinemia, also taking into account the C677T polymorphism of the gene codifying for MTHFR enzyme.

## 2. Patients and Methods

### 2.1. Study Population

From September to November 2000, about 2000 subjects were approached among the staff and student populations of the “Agostino Gemelli” University Hospital of the Catholic University, School of Medicine, and the staff of the National Institute for Research on Health and Nutrition (INRAN) in Rome, Italy.

From December 2000 to February 2001, suitable volunteers (*n* = 1240, 481 men and 759 women, aged 18–60 years) were screened for total plasma Hcy levels.

Exclusion criteria were current use of B-vitamins supplements, presence of any acute or chronic disease, pregnancy or planned pregnancy and use of medications interfering with folate metabolism. The study was approved by the Ethical Committee of the Catholic University; written informed consent was obtained from all subjects.

Subjects with Hcy levels between the 75° (Hcy = 10.0 µmol/L) and the 97° centile (Hcy = 22.2 µmol/L, *n* = 273) were invited to participate into the trial. The same group of subjects was studied for the MTHFR C677T polymorphism. One hundred fifty eight agreed to participate and 149 completed the study.

There were no significant differences in Hcy levels between the 149 subjects who completed the study and the 124 who did not accept (*n* = 115) or withdrew early (*n* = 9) (11.8 µmol/L, 10.7–13.6 *vs.* 11.7 µmol/L, 10.7–13.6, *p* < 0.7; median and interquartile range) and in the main characteristics including MTHFR genotypes. However, the number of vegetable eaters was higher in the participant group (58.0% *vs.* 44.2%, *p* < 0.05).

### 2.2. Dietary Assessment

Food consumption data were collected at individual level every 10 days using the estimated one-day food record method with a semi-structured diary. All foods and drinks consumed, both at and outside home, were recorded by each participant using household measures and estimating portion sizes according to detailed guidance notes and photographs atlas [10]. The USDA food composition database was used to provide the folate content of foods (USDA Nutrient database for standard reference).

Since our study was planned before the advent of dietary folate equivalents (DFEs), a concept introduced to account for the varying bioavailability of folates from different sources [11], target intakes and analyses are presented in the original units (μg/day), while estimated DFEs were presented in parentheses in the text.

### 2.3. Study Design

The study was a 13 week open, randomized, double blind clinical trial. For the intervention study, the following folate enrichment strategies were used:

- habitual diet (usually containing about 220 μg of natural folate) + 200 µg from natural folate-rich diet, daily; subjects were advised to consume their habitual diet plus additional natural folate-rich foods to achieve an additional intake of 200 µg/day of folate; this was obtained throughout a “score diet of at least 10 points” (1 point = 20 µg);
- habitual diet + [6*S*]5-methyltetrahydrofolate 200 µg (340 DFEs), daily;
- habitual diet + folic acid 200 µg , (340 DFEs) daily;
- habitual diet + placebo.

The nutritional intervention was open, while the pill supplements were double-blinded.

Participants were also stratified according to their MTHFR genotype (42 subjects for each genotype) before randomization to ensure the same distribution of CC, CT and TT genotype in each group.

### 2.4. Supplements

Folic acid and [6*S*]5-MTHF capsules were obtained from Merck Eprova AG (Schaffhausen, Switzerland) as hard gelatin capsules containing 208 μg (454 nmol) for the [6*S*]5-MTHF moiety), or 200 μg (454 nmol) folic (monoglutamic) acid. The randomization code of the supplements was kept at the Institute of Food Reseach, Norwich, UK (Dr. PM Finglas).

The subjects were counseled in how to enrich the diet in order to achieve 400 μg of daily folate intake, by a booklet showing the food folate content both for 100 g and per portion and suggesting the possible food combinations.

Compliance concerning the diet and the supplements was assessed by counting the pills still remaining, by phone interviews (at 4, 8 and 13 weeks).

All participants were advised do not use any other folate or vitamin supplements during the study.

Total plasma Hcy, RBC folate, vitamins B6 and B12 were determined at the beginning and at the end of the trial.

### 2.5. Specimen Collection and Biochemical Measurements

Blood samples were obtained between 08:00 AM and 10:00 AM after an overnight fast.

Serum sample was used for creatinine measurement on an automated chemistry analyzer (Roche Hitachi 917); hematocrit and red cell count were measured in tripotassium ethylendiaminetetraacetic acid (K3 EDTA) (Vacuette, Greiner Bio-One GmbH, Kremsmünster, Austria) on the Advia 120 hematology analyzer (Bayer, Terrytown, NY, USA). These samples were analyzed within two hours from collection; internal quality control samples were daily processed with sample analysis.

For total Hcy assay, whole blood samples were collected on ice-cooled tubes containing K3 EDTA and centrifuged within 30 min at 2000× *g* for 10 min at 4 °C; plasma aliquots were frozen at −80 °C until assayed. Hcy was assayed according to the HPLC method of Araki and Sako [12] with slight modifications, as reported elsewhere [13]. Intraassay and interassay coefficients of variation (CVs) were <3.1% and <4.5% respectively.

Whole blood samples, collected into K3 EDTA anticoagulated tubes, were used for RBC folate assay by using a microbiological assay according to Wright *et al.* [14]. The coefficient of variation of the assay was between 11.2% and 15.0%. Concentration of RBC folate ≥305 nmol·L^−1^ was considered adequate, according to the Institute of Medicine [11].

Vitamine B6 was measured by HPLC method with fluorimetric detection by using a commercially available kit (Chromsystems Instruments and Chemicals GmbH, Munchen, Germany), according to the manufacturer’s instructions; intra and inter-assay coefficients of variation (CV) were <4.0% and <7.0% respectively. Vitamin B12 was measured by using an immunoassay on the Abbott AxSYM analyzer (Abbott Laboratories, Abbott Park, IL, USA); the total CV was <10.0%.

Blood collected on filter paper was directly used for PCR amplification for the study of the C677T mutation of the MTHFR gene at the Department of Pediatrics, Federico II University, Napoli, Italy. Half of the blood spot was cut and submerged in 130 µL of distilled water, covered with a drop of paraffin oil, and heated at 96 °C for at least half an hour. At the end of this time, 25 µL of the PCR amplification mix were added directly to the template (25 µL of lysate). DNA amplification and restriction analysis by HinfI were carried out as previously described [15].

### 2.6. Statistical Analysis

Changes in RBC folate and Hcy concentrations between baseline and the follow-up visits were the primary variables of interest. Since their values were not normally distributed, median values with interquartile ranges (25th–75th percentiles) or geometric means and (95% Confidence Intervals) were displayed. Changes in Hcy concentrations from baseline to follow-up were calculated by analysis of variance for non-parametric analysis by Kruskal–Wallis test. Differences in pre-treatment Hcy levels among MTHFR-polymorphism groups were derived through Mann-Whitney test. To calculate the odd to reach a good response (Hcy levels <25%) after enriched diet, 5-MTHF or folic acid, as compared to placebo, multiple logistic regression analysis, adjusted for sex, BMI, MTHFR genotypes, RBC folate, vitamin B6 and vitamin B12, was performed. Results were considered significant at *p* < 0.05. (Statistical analyses were done by using Stata software, version 10.0.)

## 3. Results

The demographic characteristics of the study population and the variables measured at baseline are shown in Table 1, Table 2. The four treatment groups did not differ significantly for age, sex, body mass index, social status, prevalence of smokers or physically actives and frequency of vegetarians, or wine or coffee drinkers. Serum levels of creatinine and hematocrit were similar across the groups. No significant differences among groups were observed in concentrations of total Hcy and vitamin cofactors and in the distribution of MTHFR genotypes.

**Table 1:** Distribution of demographic, anthropometric, life-habits and genetic characteristics of participants according to treatment group ^1^.

*n* = 149	Treatment group				
	Enriched Diet (*n* = 35)	% 5-MTHF (*n* = 39)	Folic acid (*n* = 37)	Placebo (*n* = 38)	*p* ^2^
Age (years)	41 (33, 51)	41 (25, 50)	40 (28, 50)	41 (31, 51)	0.87
Sex, male (%)	54.3	59.0	62.2	57.9	0.93
BMI (kg/m^2^)	23.8 (21.6, 26.5)	23.4 (21.5, 25.9)	24.3 (21.0, 26.8)	23.7 (22.1, 25.8)	0.83
Weight (kg)	70.5 (60.5, 84.5)	66.0 (55.5, 79)	71.5 (58, 83)	67.0 (56.5, 79.0)	0.64
Height (cm)	171 (161, 177)	168 (162, 179)	172 (165, 176)	168 (160, 179)	0.95
Current smokers (%)	28.6	33.3	29.7	36.8	0.87
High educational level (%)	74.3	76.9	83.8	63.2	0.22
Health care professionals (%)	28.6	38.5	40.5	26.3	0.34
Physical active (%)	37.1	43.6	32.4	52.6	0.29
Vegetables eaters (%)	51.4	41.0	43.2	52.6	0.81
Wine drinkers (%)	51.4	69.2	56.8	65.8	0.28
Coffee drinkers (%)	22.9	30.8	40.5	26.3	0.23
MTHFR polymorphism (%)					0.98
*Homozygotes for wild-type*	42.9	33.3	32.4	34.2	
*Heterozygotes*	31.4	38.5	40.5	39.5	
*Homozygotes for variant allele*	25.7	28.2	27.1	26.3	

^1^ Continuous skewed are presented as median (1st, 3rd quartile) and categorical as frequencies; ^2^ *p*-values for differences between treatment groups derived through Pearson’s *X*^2^ for categorical variables and Kruskal–Wallis test for continuous skewed data.

**Table 2:** Baseline blood biomarkers of the participants according to treatment group ^1^.

*n* = 149	Treatment group				
	Enriched diet (*n* = 35)	% 5-MTHF (*n* = 39)	Folic acid (*n* = 37)	Placebo (*n* = 38)	*p* ^2^
Homocystein (μmol/L)	14.3 (11, 17.3)	14 (11.1, 18.1)	13.3 (11.6, 15.3)	14.8 (10.1, 17.2)	0.96
Red blood cells (millions)	4.64 (4.32, 5.15)	4.89 (4.43, 5.34)	4.91 (4.60, 5.19)	4.76 (4.43, 4.97)	0.32
Hemoglobin (g/dL)	13.5 (12.9, 14.7)	13.8 (13.0, 15.2)	14.7 (13.2, 15.1)	14.4 (13.6, 14.7)	0.42
Haematocrit (%)	42.2 (39.3, 45.9)	42.4 (40.0, 47.2)	44.6 (40.5, 46.3)	43.5 (40.5, 44.9)	0.65
Creatinine (mg/dL)	0.8 (0.7, 0.9)	0.8 (0.7, 1.0)	0.8 (0.7, 1.0)	0.8 (0.7, 0.9)	0.53
Serum B6 (ng/mL)	9.0 (6.4, 12.8)	9.3 (6.2, 10.9)	8.0 (5.1, 12.5)	8.7 (6.7, 11.9)	0.55
Serum B12 (pg/mL)	388 (329, 494) *	310 (252, 437)	307 (250, 388)	311 (225, 386)	0.04

^1^ Continuous skewed are presented as median (1st, 3rd quartile); ^2^ *p*-values for differences between treatment groups derived through and Kruskal–Wallis test for continuous skewed data; * Significantly different from the other three groups.

**Figure 1:**
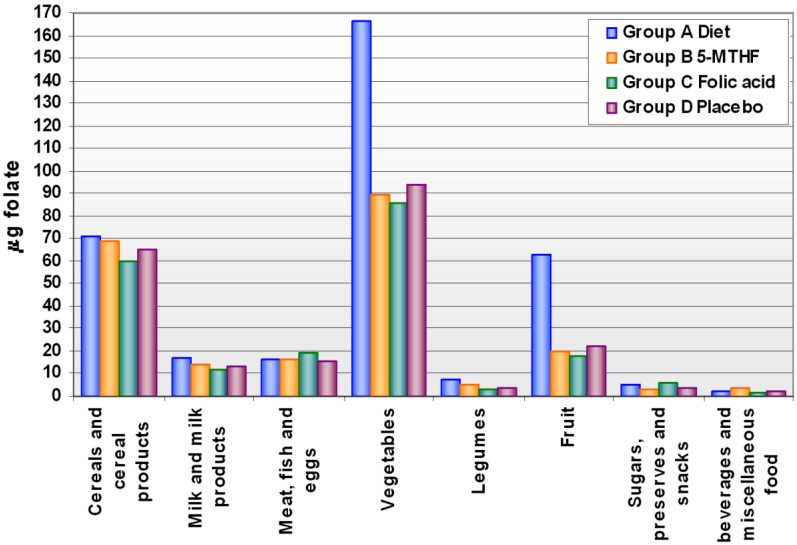
Folate intake through main food groups after supplementation, by treatment group.

Levels of folate intake at baseline and after supplementation are shown in Supplementary Table S1. The mean intake of food folate was 212.2 ± 55.1 µg/day and did not differ, at baseline, across groups. After the supplementation period, food folate intake increased by 71% after 13 weeks of a diet enriched in folate. The increase was mainly due to an increased intake of fruit and vegetables, while the intake of other food groups did not change compared to the other treatment groups (Figure 1).

**Table 3:** Distribution of Hcy levels before and after the intervention according to treatment groups.

Treatment group	Pre-treatment Homocysteine ^1^ (μmol/L)	Post-treatment Homocysteine ^1^ (μmol/L)	% Change after treatment ^2^	*p* ^3^	*p* ^4^	Homocysteine decrease >25% (%)
Enriched Diet (*n* = 35)	13.9 (12.2, 15.9)	11.1 (9.5, 12.9)	−20.1	0.002	<0.001	42.9
5-MTHF (*n* = 39)	13.9 (12.2, 15.9)	11.2 (9.8, 12.8)	−19.4	0.001	0.002	46.2
Folic acid (*n* = 37)	13.7 (12.5, 15.1)	10.7 (9.6, 11.9)	−21.9	<0.001	<0.001	48.7
Placebo (*n* = 38)	14.6 (12.7, 16.8)	15.3 (13.2, 17.7)	4.8	0.23		10.5

^1^ Pre- and post-treatment homocysteine are presented as geometric means (95% CI); ^2^ % change after treatment: The post treatment geometric mean divided by the pretreatment geometric mean; ^3^ *p*-values derived for differences in pre- and post-treatment homocysteine for each treatment group, through Wilcoxon’s test; ^4^ *p*-values derived for differences in median homocysteine change after treatment between groups of treatment and placebo scheme through, Mann–Whitney’s test.

In Table 3, changes in Hcy concentrations after the various interventions are shown. Hcy concentrations were reduced after folate enriched diet, 5-MTHF or folic acid supplementation by 20.1 (*p* < 0.002), 19.4 (*p* < 0.001) and 21.9% (*p* < 0.001) respectively, as compared to baseline levels, whereas an increase in Hcy concentrations by 4.8% (n.s.) was observed after placebo. Changes induced by interventions were significantly different from changes after placebo (*p* < 0.001, *p* < 0.002 and *p* < 0.001, respectively for enriched diet, 5-MTHF and folic acid). There were no significant differences across treatments.

We also considered the number of subjects who reached a decrease in basal Hcy levels of at least 25%. This target was reached by 42.9%, 46.2%, 48.7% and 10.5% of subjects treated respectively with enriched diet, 5-MTHF, folic acid and placebo. Multiple logistic regression analysis adjusted for sex, BMI, MTHFR genotypes, RBC folate, vitamin B6 and vitamin B12, shows an increased odd to reach a good response (Hcy levels <25%) after enriched diet, 5-MTHF or folic acid of 7.6 (3.9–42.6), 7.9 (4.2–43.0) or 8.2 (5.9–48.5), respectively, as compared to placebo.

As expected, subjects homozygotes for the MTHFR TT genotype tended to have higher, although not significant, levels of Hcy than carriers the C allele (CC and CT) among three of the treatment groups, while a reverse pattern was seen for the5-MTHF treatment group. After enriched diet and folic acid supplementation, a decrease in Hcy concentrations was observed in both genotypes although it was higher in TT homozygotes (Table 4).

**Table 4:** Distribution of Hcy levels before and after the intervention according to treatment groups and MTHFR genotypes.

Treatment group	MTHFR polymorphism	Pre-treatment Homocysteine ^1^ (μmol/L)	Post-treatment Homocysteine ^1^ (μmol/L)	% Change after treatment ^2^	*p* ^3^	*p* ^4^
Enriched Diet	*TT* (n *= 9*)	15.1 (10.6, 21.4)	11.3 (7.8, 16.3)	−25.2	0.46	0.07
	*CT/CC* (n *= 26*)	13.5 (11.7, 15.7)	11.0 (9.2, 13.2)	−18.5		0.01
5-MTHF	*TT* (n *= 11*)	12.8 (9.1, 18.2)	11.8 (8.9, 15.6)	−7.8	0.47	0.37
	*CT/CC* (n *= 28*)	14.3 (12.4, 16.5)	11.0 (9.4, 12.8)	−23.1		0.002
Folic acid	*TT* (n *= 10*)	15.3 (11.6, 20.3)	10.6 (8.1, 14.0)	−30.7	0.35	0.01
	*CT/CC* (n *= 27*)	13.2 (12.0, 14.5)	10.7 (9.4, 12.1)	−18.9		0.001
Placebo	*TT* (n *= 10*)	17.4 (13.1, 23.2)	21.0 (16.1, 27.4)	20.7	0.07	0.15
	*CT/CC* (n *= 28*)	13.7 (11.7, 16.1)	13.7 (11.6, 16.1)	0.21		0.62

^1^ Pre- and post-treatment homocysteine are presented as geometric means (95%CI); ^2^ % change after treatment: The post treatment geometric mean divided by the pretreatment geometric mean; ^3^ *p*-values for differences in Pre-treatment homocysteine levels among MTHFR-polymorphism groups derived through Mann–Whitney test.; ^4^ *p*-values derived for differences in pre- and post-treatment homocysteine for each treatment group and each MTHFR polymorphism, through Wilcoxon’s test.

After 13 weeks of folate supplementation, a statistically significant increase in mean RBC folate concentration was observed only after folic acid supplementation; while in the other two treatment groups the increase was small and not significant (Table 5). The geometric mean concentration of RBC folate increased by 9.7%, 3.8% and 16.1% after enriched diet, 5-MTHF and folic acid, respectively. Both enriched diet and folic acid intake determined a significant increase in RBC folate concentration as compared to placebo (*p* < 0.02 and 0.01, respectively) while the increase induced by 5-MTHF intake was not significant.

**Table 5:** Distribution of serum RBC levels before and after the intervention according to treatment group.

Treatment group	Pre-treatment RBC folate ^1^	Post-treatment RBC folate ^1^	% Change after treatment ^2^	*p* ^3^	*p* ^4^
Enriched Diet (*n* = 33)	225 (210, 241)	247 (223, 275)	9.7	0.12	0.02
5-MTHF (*n* = 38)	218 (205, 233)	226 (205, 250)	3.8	0.53	0.24
Folic Acid (*n* = 37)	218 (199, 239)	253 (228, 280)	16.1	0.01	0.01
Placebo (*n* = 37)	227 (211, 243)	216 (191, 243)	−4.8	0.35	

^1^ Pre- and post-treatment RBC folate are presented as arithmetic means (95% CI); ^2^ % change after treatment: The post treatment geometric mean divided by the pretreatment geometric mean; ^3^ *p*-values derived for differences in pre- and post-treatment RBC folate for each treatment group, through Wilcoxon’s test; ^4^ *p*-values derived for differences in % change of RBC folate after treatment between groups of treatment and placebo scheme, through Mann–Whitney’s test.

## 4. Discussion

Different folic acid supplementations by enriched diet (200 μg/day), 5-MTHF (200 μg/day, 340 DFE), or folic acid (200 μg/day, 340 DFE), induced a significant reduction in Hcy concentrations in comparison with the control group.

The efficacy of 5-MTHF has been compared with that of folic acid in several studies with contrasting results: Fohr *et al.* [16] showed that in women folic acid was more effective than MTHF in lowering plasma total Hcy, while Venn *et al.* [17] reported that a low-dose of 5-MTHF was at least as effective as folic acid in reducing total Hcy concentrations in healthy subjects. In contrast, 5-MTHF was more effective than folic acid in increasing plasma folate levels in the study of Prinz-Langenohl [18] both in TT and CC subjects.

The decrease in Hcy concentration was only partially influenced by the MTHFR genotype; indeed enriched diet and folic acid were more effective in decreasing Hcy levels in TT homozygotes as compared with carriers of C allele. Our data partially agree with those of Fohr *et al.* [16] who reported the most pronounced total Hcy-lowering effect occurring in women with the TT genotype. The smaller percent change in Hcy observed in the TT subjects treated with 5-MTHF, could be due to the low baseline value rather than the genotype. However, the small number of subjects with different genotypes in each treatment group should be considered.

We demonstrated that an adequate folate status could also be achieved by eating a greater amount of natural folate-rich foods, even if the bioefficacy of folate from some foods is significantly lower than that of folic acid itself; the bioefficacy of natural folate, in fact, is closely dependent on the food matrix and on other factors. However, bioefficacy of folate derived from vegetables and citrus fruit ranges from 60% to about 100% of that of folic acid [8,19]. In our study, the enrichment of diet with folate was mainly obtained by introducing more fruit and vegetables, while the consumption of other food groups rich in folate was similar to the other supplementation groups.

A limitation of our study is the use of RBC folate concentration to evaluate the folate status; indeed, although it is usually considered a better index of body folate stores, the short duration of the intervention period, makes RBC folate concentration only partially reflecting post-supplementation folate status. This could explain the small changes in RBC folate concentration observed after treatment. Our results show a stronger effect in increasing RBC folate concentration of folic acid (+16.1%), followed by natural folate enriched diet (+9.7%) whereas a lower percent variation was observed with the 5-MTHF treatment (+3.8%). These data are apparently at variance with those of other studies, which used, however, different supplementation protocols: in the study of Fohr *et al.* [16] (FA and 5-MTHF 400 µg/day, for 8 weeks), the post-treatment variation in RBC folate was +63.4% and +59.7%, respectively. Cuskelly *et al.* [20] found a variation of +40.2% and +52% after supplementation with 400 µg/day of folic acid and fortified foods respectively for 12 weeks; the same authors, in the group supplemented with 400 µg/day of natural folate enriched diet, found a lower increase in the concentration of RBC folate (+11%). In addition, in the study of Venn [17], after a lower supplementation (folic acid and 5-MTHF 100 µg/day, for 24 weeks), the percentage of variation after 24 weeks of treatment was +31% and +23%, respectively.

In our study, daily folate enrichment of 200 μg/day of the usual diet (containing about 220 μg/day) with the three strategies adopted (increased consumption of folate rich foods, pill of 5-MTHF, pill of folic acid) resulted effective in lowering significantly serum Hcy, however only folic acid supplementation was able to increase RBC folate in subjects with mild hyperhomocysteinemia. Mean typical folate intake in Italy is 220 µg/day, with a suggested minimum intake of 200 µg/day. Increasing folate intake to 400 µg/day achieves near-maximal Hcy reduction and has been proposed as the minimum recommended folic acid intake for NTDs (neural tube defects) prevention [21]. In Italy, fortification with folic acid is not mandatory, although there is an increasing trend for food manufacturers to voluntarily fortify breakfast cereals, which are not frequently used by the general population [22]. We planned our study to increase the daily intake of folate to the optimal level of 400 µg/day by adding to the usual dietary intake foods rich in folate, or two different pharmacological forms of folic acid. Our study demonstrated that, increasing the consumption of fruit and vegetable is effective in decreasing Hcy concentrations in a comparable extent to the pharmacological supplementations. Our data are in agreement with other studies showing that an increase in folic acid intake up to 200 µg/day may result as effective in reducing tHcy concentration as supplementation at higher levels [23,24]. The majority of the studies that investigated about folate intake and Hcy concentration, used pharmacological supplementation (folic acid, 5-MTHF) or folic acid fortified foods; not many studies considered the effects of natural folate enriched diet [25,26,27]. Our data partially agree with those obtained by Ahfield-Watt *et al.* [25] which showed a similar Hcy lowering effect of folate enriched diet (−14%) and folic acid supplements (−16%); the post-treatment variation in folate status is not easily comparable because these authors measured levels of folate only in plasma. It is however interesting to note that a significant increase in folate levels and a significant reduction in Hcy concentrations were obtained also with the enriched diet, although the enriched diet in this study included not only natural folate but also folic acid fortified foods. Therefore, a folate rich-diet could be suggested to free-living population to maintain an adequate folate status, also taking into account that it provides, beside folates, other healthy nutrients such as antioxidants and other vitamins [20].

Although our results show that the supplementation with natural folate rich foods, in a free-living mediterranean population, reached a reduction in Hcy concentrations, comparable to that obtained with pharmacological supplements (folic acid and 5-MTHF), it’s difficult to imagine that this could be the best and simplest strategy to reach and to maintain for a longer period, an adequate folate status; the pharmacological supplementation certainly assures more prompt effects especially in particular conditions such as pregnancy planning, in which a rapid improvement in folate status is desirable to prevent adverse pregnancy outcome and NTDs.

## 5. Conclusions

Our results show that the supplementation with natural folate rich foods, folic acid and 5-MTHF in a free-living mediterranean population, reached a similar reduction in Hcy concentrations. However, it could be advisable to prefer a pharmacological supplementation in particular conditions such as pregnancy.

## Supplementary

**Table S1:** Food folate intake at baseline and after supplementation, by treatment groups.

Folate intake μg/day	Diet *n* = 35	5-MTHF *n* = 39	Folic acid *n* = 37	Placebo *n* = 38
**Baseline**	205.2 ± 54.9	220.5 ± 44.9	202.5 ± 66.8	220.5 ± 53.9
**After supplemetation**	350.9 ± 76.1	237.2 ± 75.5	236.2 ± 75.7	218.5 ± 88.3
